# Socioeconomic status in the association between use of personal care products and exposure to endocrine-disrupting chemicals in pregnant Taiwanese women

**DOI:** 10.3389/fpubh.2025.1537669

**Published:** 2025-04-10

**Authors:** Alexander Waits, Chia-Huang Chang, Yu-Fang Huang, Ming-Song Tsai, Jia-Woei Hou, Pei-Wei Wang, Chih-Yao Chen, Chia-Jung Hsieh, Ming-Tsang Wu, Shu-Li Wang, Mei-Lien Chen

**Affiliations:** ^1^School of Medicine, Institute of Public Health, National Yang Ming Chiao Tung University, Taipei, Taiwan; ^2^School of Public Health, Taipei Medical University, Taipei, Taiwan; ^3^School of Medicine, Institute of Environmental and Occupational Health Sciences, National Yang Ming Chiao Tung University, Taipei, Taiwan; ^4^Department of Obstetrics and Gynecology, Cathay General Hospital, Taipei, Taiwan; ^5^Department of Pediatrics, Cathay General Hospital, Taipei, Taiwan; ^6^Department of Pediatrics, Taipei City Hospital, Taipei, Taiwan; ^7^Department of Obstetrics and Gynecology, Division of Obstetrics and High-Risk Pregnancy, Taipei Veterans General Hospital, Taipei, Taiwan; ^8^Department of Public Health, Tzu Chi University, Hualien, Taiwan; ^9^Research Center for Environmental Medicine, Kaohsiung Medical University, Kaohsiung, Taiwan; ^10^Division of Environmental Health and Occupational Medicine, National Health Research Institutes, Zhunan, Taiwan

**Keywords:** personal care products, bisphenol A, parabens, income, pregnancy, socioeconomic status

## Abstract

**Background:**

Maternal exposure to endocrine-disrupting chemicals (EDCs), particularly those found in personal care products (PCPs), may affect child development. Socioeconomic inequalities in EDC exposure warrant further investigation. This study assessed the role of income and education in the association between PCP use and exposure to bisphenol A (BPA) and parabens in pregnant women.

**Methods:**

Associations between PCP use and urinary concentrations of BPA and four parabens in pregnant women from the Taiwan Maternal and Infant Cohort Study were estimated using linear regression, with results expressed as the percentage change in concentrations for each additional PCP use per week. The analysis was stratified by income and education and predicted concentrations, and a 95% confidence interval (CI) was graphed according to the frequency of PCP use.

**Results:**

Higher concentrations of methylparaben, ethylparaben, and propylparaben were associated with more frequent use of different PCPs, especially makeup. The above-lowest income group showed positive associations between frequency use of rinse-off PCPs and methylparaben (2.5%, 95%CI = 0.9%, 4.0%), propylparaben (2.8%, 95%CI = 0.3%, 5.3%), and between leave-on PCPs and methylparaben (3.1%, 95%CI = 1.8%, 4.4%), ethylparaben (2.2%, 95%CI = 0.1%, 4.2%), and propylparaben (2.8%, 95%CI = 0.8%, 4.9%). BPA was negatively associated with rinse-off PCPs (−1.2%, 95%CI = −2.3%, −0.2%). A positive association between leave-on PCPs and BPA was suggested in the lowest income group (1.7%, 95%CI = −0.4%, 3.7%). Predicted BPA concentrations were significantly higher in the lowest income group at higher frequencies of PCP use. Stratification by education showed the strongest associations in the postgraduate group for rinse-off PCPs with methylparaben (6.1%, 95%CI = 1.9%, 10.5%) and propylparaben (6.9%, 95%CI = 1.2%, 12.9%), as well as for leave-on PCPs with methylparaben (4.1%, 95%CI = 1.2%, 7.2%).

**Conclusion:**

The associations observed between various PCPs and parabens suggest that reducing the use of certain PCPs in pregnant women could help lower paraben exposure. Higher levels of BPA in the lowest income group require further investigation of sources of BPA exposure, especially in disadvantaged populations.

## 1 Introduction

Personal care products (PCPs) are known sources of exposure to endocrine-disrupting chemicals (EDCs) such as parabens, bisphenols, and others. Parabens are commonly found in PCPs, such as shampoos, moisturizers, shaving gels, personal lubricants, topical pharmaceuticals, makeup, and toothpaste, and are also used as food preservatives (1, 2). Human exposure to bisphenol A (BPA) is mainly via food and drinking water (3, 4); however, more recent studies reported substantial levels of BPA in a wide range of PCPs (5).

The growing societal concern over EDCs has become prominent in the past decade, especially regarding vulnerable populations (6–8). For example, a number of studies linked chemical exposure via PCPs to the disproportionate asthma burden in the US black population (9). Few studies explicitly examined socioeconomic status (SES) and EDC levels, with inconsistent support for the environmental justice hypothesis that poorer populations are more exposed to pollutants. Specifically, Americans with lower income had higher levels of BPA and phthalates, but lower levels of polyfluoroalkyl substances (6, 10). Family income was by far the most consistent and important predictor of BPA concentrations with a clear dose–response pattern (6); however, no studies explicitly focused on the role of income/education in the association between PCP use and BPA/paraben exposure. Pregnant women, in particular, are of significant interest due to the potential risks EDCs pose to both maternal and fetal health; however, research on the relationship between EDC exposure and socioeconomic factors in pregnant women remains limited (11). Specifically, PCP use, as a source of EDC exposure, has been shown to vary among pregnant women based on ethnicity, maternal education, and insurance status (12).

Our previous analysis identified some weak associations between socioeconomic factors and EDC concentrations but it also highlighted considerable inequalities in BPA and paraben levels among pregnant women (13). In this study, we aimed to identify groups of pregnant women who may be particularly susceptible to high BPA and paraben exposure due to PCP use. We did this by estimating the associations between PCP use frequency and BPA/paraben concentrations, while also assessing the role of income and education in these associations.

## 2 Materials and methods

We utilized the data from the Taiwan Maternal and Infant Cohort Study (TMICS) collected during 2012–2016 at nine hospitals in the North, Central, South, and East regions of Taiwan. A detailed cohort description has been published (14). Briefly, pregnant women were enrolled during their routine third-trimester antenatal examinations, weeks 29–40. Women with a history of systemic diseases (e.g., cancer, hypertension, or diabetes), chronic use of corticosteroids or immunosuppressants, or aged over 45 years, were excluded. Participants provided urine samples for the analysis of EDC metabolites and completed a questionnaire. We excluded individuals with invalid (e.g., mistyped or out-of-range) or missing values in questions related to PCP use, household income, education, working status, or incomplete laboratory data. All pregnancies included in this study were uncomplicated.

The frequency of PCP use was assessed for rinse-off (body wash, shampoo, facial cleanser, and hand soap) and leave-on products (lotion, toner, lip balm, makeup, essential oil, perfume, and hair spray). Based on seven discrete categories of use frequency in the questionnaire, we standardized responses to express frequencies as times per week (Supplementary Table S1). Furthermore, we dichotomized PCP use based on the distributions into ever vs. never (essential oil, perfume, and hair spray) and less than four times per week versus four times per week or more (body wash, shampoo, facial cleanser, hand soap, lotion, toner, lip balm, and makeup).

Income was reported in Taiwanese dollars (100 NTD≈3.1 USD) in six discrete categories. Due to the small number of observations in some categories and the lack of significant differences among higher income groups, we dichotomized income using a cutoff of 0.5 million NTD (approximately twice the minimum salary in Taiwan during the study period). Participants were categorized into the lowest income group (< 0.5 million NTD) and the above-lowest income group (≥0.5 million NTD), which allowed us to define the most underprivileged group. Education was categorized as (1) high school or lower, (2) college, and (3) postgraduate. Other confounders included marital status, employment, region of residence, and pre-pregnancy body mass index (BMI). Given the differences in BMI-health risk associations between Asian and European populations (15), we used locally developed BMI cutoffs of 18, 24, and 27 kg/m^2^ to categorize underweight, normal, overweight, and obese women (16).

We analyzed the concentrations of urinary metabolites of BPA and four parabens (methylparaben, ethylparaben, and butylparaben). Details of analytical methods were previously published for BPA (17) and parabens (18). Since concentration distributions were highly skewed, concentrations below the limit of detection (LOD) have been treated as half the LOD value (19).

### 2.1 Statistical analysis

**Figure 1:**
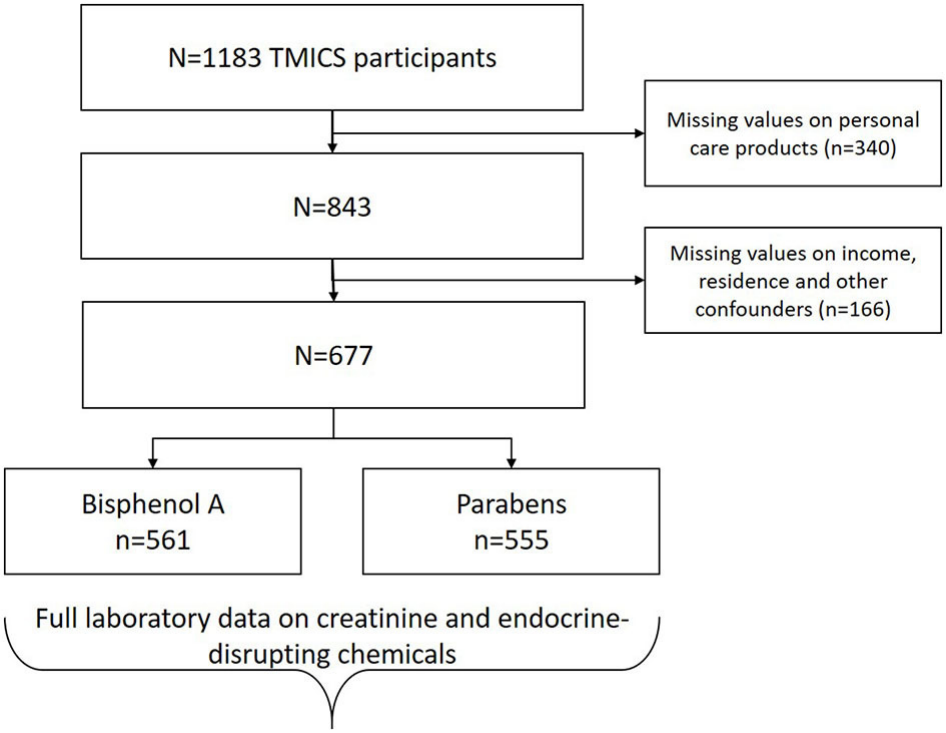
Flowchart of sample selection.

Observations with missing values on PCP use, income, and education were case-wise excluded from the analysis (Figure 1) and compared to those without missing values on age and regions (Supplementary Table S2). Socio-demographic variables and PCP use were compared between income/education groups using the analysis of variance test, an independent *t*-test for continuous variables, and a chi-square test for categorical variables. EDC concentrations were analyzed in ng/mL and adjusted for molar weight and creatinine (nmol/g creatinine). Distributions were compared using the Mann–Whitney U-test for differences between the two income groups and between low- and high-frequency PCP users. The Kruskal–Wallis test was used to compare distributions across the three education groups. Sensitivity analyses were conducted by excluding extreme outliers for BPA (*n* = 1) and methylparaben (n=10), which did not change the results.

Adjusted EDC concentrations were ln-transformed to approximate a normal distribution and fit into linear regression models as dependent variables. Linear regression models were adjusted for age, income, education, BMI, working status, and geographical region. Tests for collinearity showed negligible variance inflation factors (VIFs) of < 1.2. Independent variables included the frequency of use of each PCP type as times/week. Similarly, linear regression models were fit for the frequency use of rinse-off PCPs and leave-on PCPs as continuous (times/week) and categorical (frequent vs. non-frequent users) and further stratified by two income groups and by three education groups. The corresponding regression coefficients and 95% confidence interval (CI) were back-transformed [100 × (*e*^β^ – 1)] to obtain percent changes in EDC concentrations. Predicted concentrations with a 95% CI were calculated for rinse-off and leave-on PCPs by the exponentiation of the predicted ln-transformed values and plotted as a function of frequency use of rinse-off and leave-on PCPs stratified by income and education groups. The moderating role of income and education was assessed by the visual examination of the overlapping 95% CI. Statistical significance was set at α = 0.05. All statistical analyses were conducted using R version 4.2.2 (R Foundation for Statistical Computing, Vienna, Austria).

## 3 Results

After excluding participants with missing values on PCP use, income, and other confounders, we obtained 677 observations, of which those with full laboratory data on creatinine and BPA (*n* = 561) and parabens (*n* = 555) were analyzed (Figure 1). Participants in the lowest income group were significantly younger and had higher percentages of those with high school education or lower (30.2% vs. 8.6%), housewives or unemployed (54.7% vs. 21.4%), residing in the South (26.6% vs. 14.7%), and the East regions (44.6% vs. 26.6%) compared to the above-lowest income group. BMI groups differed significantly, with the lowest income group having higher percentages of overweight (17.3% vs. 11.2%) and obese (12.9% vs. 8.0%) and a lower percentage of underweight (10.1% vs. 14.8%) compared to the above-lowest income group. College and postgraduate education groups were older, had higher percentages of employed, and were living in the North and Central regions. The lowest income group had significantly lower frequency use of leave-on PCPs (15.9±15.1 vs. 21.0±16.1 times per week). The lowest income group had significantly less frequent use of rinse-off PCPs (hand soap) and leave-on PCPs (lotion, toner, makeup, essential oil, and hair spray). The lower education group had more frequent use of rinse-off PCPs (body wash) but less frequent use of leave-on PCPs (makeup and essential oil) (Table 1).

**Table 1:** Distribution of demographic characteristics and use of personal care products in pregnant women across socioeconomic factors.

	**Total**	**Annual household income**			**Education**			
	**(*****N*** = **677)**	<**0.5 million NTD**^a^ **(*****n*** = **139)**	≥**0.5 million NTD**^a^ **(*****n*** = **538)**	***p*** **-value** ^b^	**High school or lower (*****n*** = **88)**	**College (*****n*** = **483)**	**Postgraduate (*****n*** = **106)**	***p*** **-value** ^b^
Age, years, mean (SD)	32.1 (4.3)	30.3 (5.1)	32.5 (4.0)	**< 0.001**	30.5 (5.9)	32.0 (4.1)	33.7 (3.2)	**< 0.001**
**Education**								
High school or lower	88 (13.0%)	42 (30.2%)	46 (8.6%)	**< 0.001**	-	-	-	-
College (undergraduate)	483 (71.3%)	97 (69.8%)	382 (71.7%)		-	-	-	-
Postgraduate	106 (15.7%)	0 (0%)	106 (19.7%)		-	-	-	-
**Marital status**								
Married	655 (96.8%)	131 (94.2%)	524 (97.4%)	0.109	82 (93.2%)	470 (97.3%)	103 (97.2%)	0.129
Single	22 (3.2%)	8 (5.8%)	14 (2.6%)		6 (6.8%)	13 (2.7%)	3 (2.8%)	
**Working status**								
Employed	486 (96.7%)	63 (45.3%)	423 (78.6%)	**< 0.001**	36 (40.9%)	358 (74.1%)	92 (86.8%)	**< 0.001**
Housewife/unemployed	191 (28.2%)	76 (54.7%)	115 (21.4%)		52 (59.1%)	125 (25.9%)	14 (13.2%)	
**Regions**								
North	119 (17.6%)	10 (7.2%)	109 (20.3%)	**< 0.001**	3 (3.4%)	80 (16.6%)	36 (34.0%)	**< 0.001**
Central	238 (35.2%)	30 (21.6%)	208 (38.7%)		25 (28.4%)	178 (36.9%)	35 (33.0%)	
South	115 (17.0%)	37 (26.6%)	78 (14.7%)		18 (20.5%)	86 (17.8%)	11 (10.4%)	
East	205 (30.3%)	62 (44.6%)	143 (26.6%)		42 (47.7%)	139 (28.8%)	24 (22.6%)	
**Pre-pregnancy body-mass index**								
Underweight, < 18.5 kg/m^2^	90 (13.3%)	14 (10.1%)	80 (14.8%)	**0.025**	50 (56.8%)	319 (66.0%)	63 (59.4%)	0.144
Normal, 18.5–24 kg/m^2^	432 (63.8%)	83 (59.7%)	355 (66.0%)		11 (12.5%)	40 (8.3%)	6 (5.7%)	
Overweight, 24–27 kg/m^2^	79 (11.7%)	24 (17.3%)	60 (11.2%)		15 (17.0%)	47 (9.7%)	17 (16.0%)	
Obese, ≥27 kg/m^2^	57 (8.4%)	18 (12.9%)	43 (8.0%)		11 (12.5%)	64 (13.3%)	15 (14.2%)	
**Frequency use of personal care products, times/week, mean (SD)**								
All products	46.7 (24.2)	41.5 (26.3)	48.1 (23.5)	**0.008**	43.4 (25.8)	46.8 (23.2)	49.1 (27.0)	0.321
Rinse-off products	26.8 (13.8)	25.7 (16.9)	27.1 (12.9)	0.368	27.7 (17.6)	26.7 (13.2)	26.2 (12.6)	0.816
Body wash	6.06 (3.87)	6.56 (4.86)	5.93 (3.56)	0.154	7.22 (5.07)	5.97 (3.63)	5.53 (3.65)	**0.035**
Shampoo	6.66 (4.54)	7.06 (5.13)	6.55 (4.37)	0.283	7.49 (5.37)	6.64 (4.40)	6.05 (4.33)	0.131
Facial cleanser	7.98 (4.62)	7.72 (5.06)	8.05 (4.51)	0.489	8.18 (5.62)	8.03 (4.41)	7.59 (4.68)	0.638
Hand soap	6.08 (7.29)	4.33 (6.18)	6.53 (7.49)	**< 0.001**	4.79 (6.62)	6.09 (7.28)	7.08 (7.76)	0.084
Leave-on products	20.0 (16.0)	15.9 (15.1)	21.0 (16.1)	**< 0.001**	15.7 (16.2)	20.1 (15.1)	22.9 (18.9)	**0.016**
Lotion	0.32 (1.45)	0.12 (0.68)	0.37 (1.59)	**0.006**	0.25 (0.97)	0.23 (1.00)	0.77 (2.82)	0.155
Toner	6.16 (5.21)	5.27 (5.28)	6.39 (5.17)	**0.026**	4.93 (5.60)	6.31 (5.05)	6.50 (5.46)	0.082
Lip balm	0.77 (2.51)	0.71 (1.89)	0.79 (2.65)	0.677	0.75 (1.96)	0.834 (2.59)	0.495 (2.57)	0.471
Makeup	5.97 (5.43)	4.62 (5.19)	6.32 (5.44)	**< 0.001**	4.44 (5.01)	6.01 (5.36)	7.10 (5.83)	**0.003**
Essential oil	3.58 (5.60)	2.71 (4.71)	3.80 (5.79)	**0.021**	2.51 (4.68)	3.56 (5.51)	4.55 (6.54)	**0.038**
Perfume	0.31 (1.66)	0.18 (0.96)	0.34 (1.79)	0.164	0.40 (2.36)	0.25 (1.16)	0.51 (2.60)	0.507
Hair spray	2.85 (4.17)	2.25 (3.98)	3.01 (4.21)	**0.049**	2.47 (4.06)	2.90 (4.09)	2.96 (4.61)	0.633

^a^One New Taiwanese Dollar (NTD) ≈ 0.031 United States Dollar.

^b^p-values for continuous variables were obtained from the analysis of variance test or independent t-test, and p-values for categorical values were obtained from the chi-square test. p-values < 0.05 bolded.

**Figure 2:**
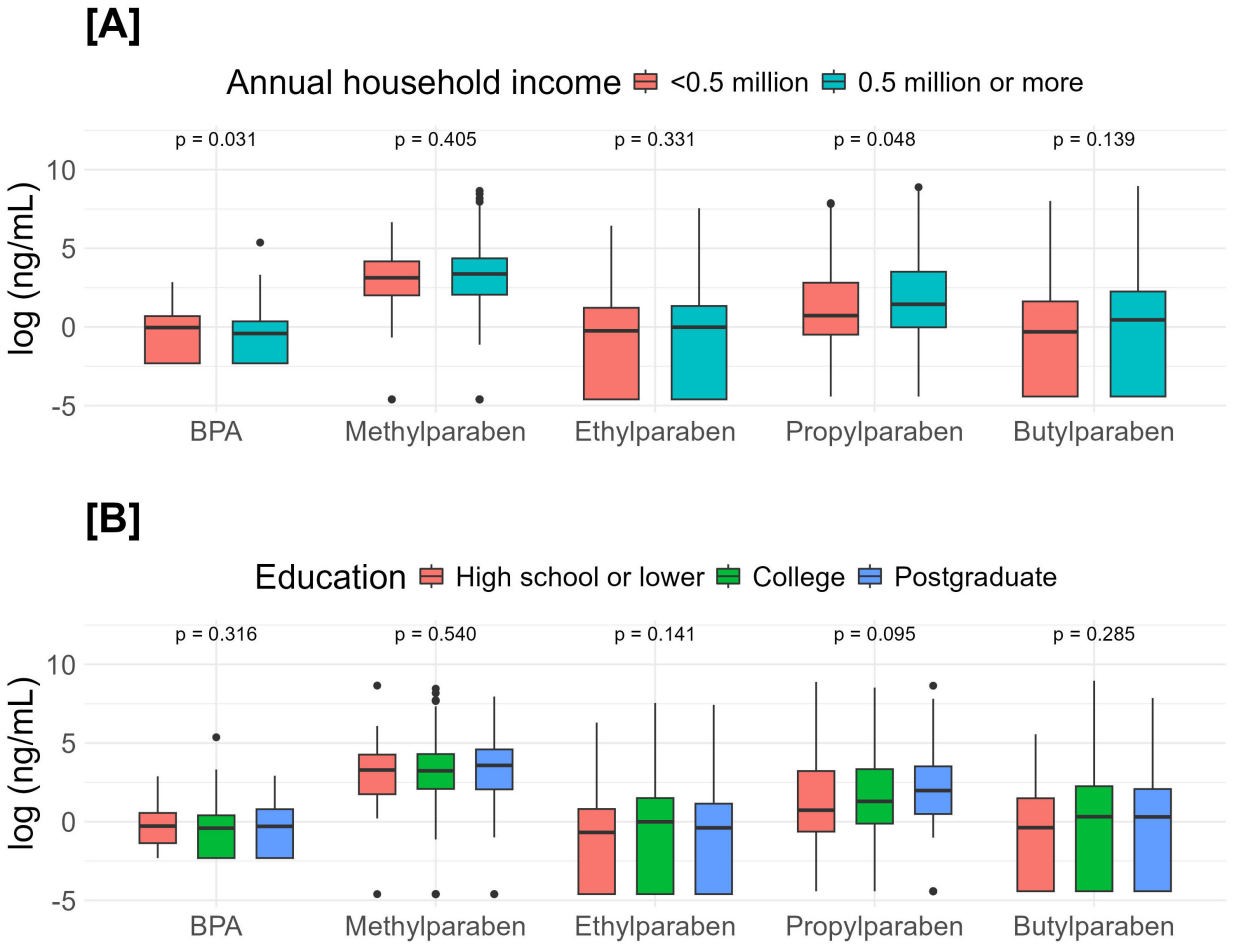
Comparisons of endocrine-disrupting chemicals urinary concentrations between income **(A)** and education **(B)**. Income groups were compared with the Mann–Whitney U-tests, and education groups were compared with the Kruskal–Wallis tests.

BPA concentration in the lowest income group (median [IQR] = 0.985 [0.099, 1.98] ng/mL) was significantly (*p* = 0.031) higher than in the above-lowest income group (median [IQR] = 0.660 [0.099, 1.45] ng/mL). Of the four parabens, only propylparaben concentration in the lowest income group (median [IQR] = 2.07 [0.659,16.9] ng/mL) was significantly (*p* = 0.048) lower than in the above-lowest income group (median [IQR] = 4.18 [0.988,32.7] ng/mL) (Table 2, Figure 2).

**Table 2:** Distributions [median (interquartile range), ng/mL] of endocrine disrupting chemicals urinary concentrations.

	**LOD**	**% < LOD**	**Total**	**Annual household income**			**Education**			
				<**0.5 million NTD**^a^	≥**0.5 million NTD**^a^	**Mann–Whitney U-test** ***p*****-value**	**High school or lower**	**College**	**Postgraduate**	**Kruskal–Wallis test** ***p*****-value**
**Bisphenol A**			***N*** **=** **561**	***n*** **=** **104**	***n*** **=** **457**		***n*** **=** **61**	***n*** **=** **410**	***n*** **=** **90**	
	0.198	32.4	0.683 [0.099,1.62]	0.985 [0.099,1.98]	0.660 [0.099,1.45]	**0.031**	0.769 [0.350,1.86]	0.668 [0.099,1.51]	0.754 [0.099,2.26]	0.316
**Parabens**			***N*** **=** **555**	***n*** **=** **102**	***n*** **=** **453**		***n*** **=** **60**	***n*** **=** **408**	***n*** **=** **87**	
Methylparaben	0.020	2.7	28.2 [7.57,76.4]	25.1 [7.46,67.3]	29.1 [7.61,79.3]	0.405	26.7 [5.75,71.2]	26.1 [8.04,73.3]	35.8 [7.82,99.0]	0.540
Ethylparaben	0.020	32.3	0.911 [0.010,3.99]	0.785 [0.01,3.42]	0.982 [0.01,4.03]	0.331	0.506 [0.010,2.24]	0.990 [0.010,4.49]	0.676 [0.010,3.15]	0.141
Propylparaben	0.024	14.8	3.87 [0.90,28.5]	2.07 [0.659,16.9]	4.18 [0.988,32.7]	**0.048**	2.08 [0.53,25.1]	3.65 [0.885,28.9]	7.24 [1.65,33.8]	0.095
Butylparaben	0.024	32.3	1.31 [0.012,9.13]	0.74 [0.012,5.10]	1.57 [0.012,9.55]	0.139	0.69 [0.012,4.44]	1.35 [0.012,9.52]	1.35 [0.012,8.01]	0.285

^a^One New Taiwanese Dollar (NTD) ≈ 0.031 United States Dollar.

LOD, limit of detection, p-value < 0.05 bolded.

**Figure 3:**
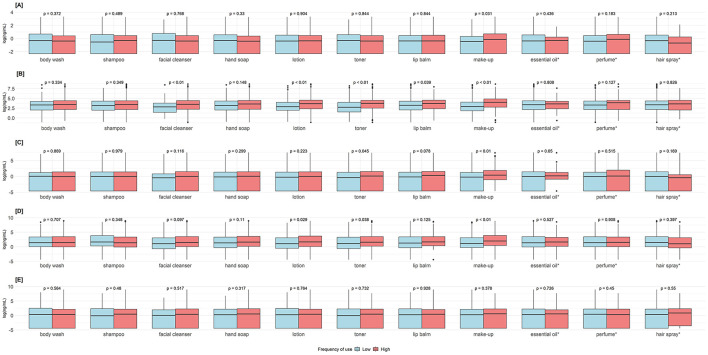
Comparisons of endocrine-disrupting chemicals urinary concentrations between low- and high-frequency users of personal care products. *P*-values obtained from Mann–Whitney U-test. Groups were dichotomized as low <4 times/week vs. high ≥ 4 times/week, except *essential oil, perfume, and hair spray as never vs. ever used. **(A)** Bisphenol A. **(B)** Methylparaben. **(C)** Ethylparaben. **(D)** Propylparaben. **(E)** Butylparaben.

Frequent users of makeup had higher concentrations of BPA (*p* = 0.031), methylparaben, ethylparaben, and propylparaben (*p* < 0.001). Frequent users of facial cleanser, lotion, and toner had higher methylparaben concentrations (*p* < 0.001). Frequent users of lotion and toner users had higher ethylparaben concentrations (*p* < 0.001) (Figure 3). Adjusted estimates for BPA showed a negative association with frequent use of body wash −26.3% (95%CI = −44.7%, −1.7%). Adjusted estimates for makeup expressed as continuous (times/week) and categorical (< 4 times/week vs. ≥4 times/week) showed positive associations with methylparaben (continuous 9.3%, 95%CI = 4.7%, 14.2%, categorical 123.2%, 95%CI = 51.7%, 228.4%), ethylparaben (continuous 7.3%, 95%CI = 0.2%, 15.0%, categorical 112.2%, 95%CI = 18.7%, 279.3%), and propylparaben (continuous 11.2%, 95%CI = 3.6%, 19.3%, categorical 127.2%, 95%CI = 25.6%, 311.1%). Adjusted estimates for lotion and toner as continuous and categorical showed consistent positive associations with methylparaben and propylparaben (Table 3).

**Table 3:** Associations between use of personal care products and urinary concentrations of endocrine-disrupting chemicals.

	**Bisphenol A**	**Parabens**			
		**Methylparaben**	**Ethylparaben**	**Propylparaben**	**Butylparaben**
	**% change (95% CI)**	**% change (95% CI)**	**% change (95% CI)**	**% change (95% CI)**	**% change (95% CI)**
**Body wash**					
Continuous^a^	−2.0 (−4.9, 1.1)	1.9 (−2.7, 6.8)	−1.3 (−8.2, 6.1)	2.0 (−5.2, 9.9)	−7.0 (−13.9, 0.4)
Categorical^b^	**−26.3 (−44.7**, −1.7)^*^	16.9 (−24.6, 81.3)	−3.8 (−49.9, 84.8)	22.3 (−37.3, 138.4)	−27.8 (−63.9, 44.4)
**Shampoo**					
Continuous	1.5 (−1.2, 4.2)	2.0 (−2.0, 6.2)	−2.4 (−8.4, 3.9)	−0.1 (−6.4, 6.5)	−1.9 (−8.3, 4.9)
Categorical^b^	−4.3 (−28.2, 27.6)	**60.5 (4.0, 147.9)** ^*^	21.2 (−36.8, 132.4)	21.3 (−37.6, 135.6)	52.4 (−23.6, 203.9)
**Facial cleanser**					
Continuous	−1.3 (−3.8, 1.3)	**4.8 (0.9, 8.9)** ^**^	5.3 (−0.8, 11.8)	6.0 (−0.4, 12.7)	1.7 (−4.6, 8.4)
Categorical^b^	−18 (−41.9, 15.8)	59.4 (−6.1, 170.5)	48.9 (−32.3, 227.6)	78.7 (−20.2, 300.1)	9.5 (−52.7, 153.7)
**Hand soap**					
Continuous	−1.1 (−2.8, 0.5)	2.1 (−0.3, 4.6)	2.5 (−1.3, 6.5)	**4.7 (0.6, 8.8)** ^*^	2.9 (−1.2, 7.2)
Categorical^b^	−11.5 (−30.6, 12.9)	26.5 (−12.6, 83.1)	31.5 (−24.2, 128.3)	53.4 (−12.6, 169.4)	21.8 (−32.2, 119)
**Lotion**					
Continuous	−0.3 (−2.6, 2)	**6.2 (2.7, 9.8)** ^***^	3.9 (−1.5, 9.5)	**6.5 (0.9, 12.4)** ^*^	1.2 (−4.4, 7.0)
Categorical^b^	4.1 (−19.5, 34.8)	**104.0 (38.4, 200.7)** ^***^	58.1 (−11.7, 183.3)	**84.5 (1.7, 234.8)** ^*^	17.2 (−37.1, 118.3)
**Toner**					
Continuous	1.0 (−1.2, 3.3)	**6.6 (3.1, 10.2)** ^***^	4.4 (−0.9, 10.0)	5.0 (−0.5, 10.8)	2.1 (−3.5, 8.0)
Categorical^b^	3.5 (−19.7, 33.4)	**96.1 (33.9, 187.2)** ^**^	68.7 (−4.9, 199.3)	**104.2 (13.8, 266.4)** ^*^	−11 (−51.7, 64.1)
**Lip balm**					
Continuous	1.3 (−0.8, 3.5)	1.3 (−1.8, 4.5)	2.4 (−2.4, 7.5)	1.1 (−3.8, 6.3)	−0.3 (−5.4, 5.0)
Categorical^b^	23.0 (−4.9, 59.1)	22.5 (−17.1, 81.2)	43.5 (−19.8, 156.7)	47.7 (−18.5, 167.7)	1.3 (−45.5, 88.3)
**Makeup**					
Continuous	1.6 (−1.4, 4.7)	**9.3 (4.7, 14.2)** ^***^	**7.3 (0.2, 15.0)** ^*^	**11.2 (3.6, 19.3)** ^**^	1.7 (−5.5, 9.5)
Categorical^b^	23.0 (−5.0, 59.2)	**123.2 (51.7, 228.4)** ^***^	**112.2 (18.7, 279.3)** ^*^	**127.2 (25.6, 311.1)** ^**^	4.9 (−43.6, 95.3)
**Essential oil**					
Continuous	−3.7 (−12.6, 6.1)	6.4 (−8.0, 22.9)	0.2 (−20.0, 25.6)	23.5 (−2.0, 55.6)	9.3 (−14.1, 39.0)
Categorical^c^	3.0 (−28.8, 49)	−5.3 (−45.9, 66.0)	−25.7 (−67.7, 71.2)	−33.4 (−71.6, 56.4)	−20.1 (−67.1, 94.2)
**Perfume**					
Continuous	1.6 (−3.4, 6.9)	**10.1 (2.3, 18.6)** ^**^	**12.8 (0.5, 26.6)** ^*^	11.9 (−0.6, 26.0)	3.1 (−8.9, 16.6)
Categorical^c^	−21.2 (−42.2, 7.4)	**−37.7 (−61.1**, −0.3)^*^	−31.6 (−66.1, 37.9)	−33.7 (−67.6, 35.9)	14.3 (−45.9, 141.2)
**Hair spray**					
Continuous	−4.6 (−11.2, 2.4)	7.1 (−3.6, 18.9)	−8.6 (−22.4, 7.6)	−2.7 (−17.8, 15)	10 (−7.6, 30.9)
Categorical^c^	28.9 (−19, 105.3)	−23.8 (−62.2, 53.9)	50.7 (−47, 328.6)	50.9 (−48.2, 339.7)	−34.3 (−78.4, 99.8)

^a^Continuous frequency use was measured as times per week.

^b^ < 4 times/week as reference vs. ≥4 times/week.

^c^Never as a reference vs. ever used.

Linear regression models were adjusted for age, income, education, body-mass index, working status, and geographical region.

^*^p < 0.05, ^**^p < 0.01, ^***^p < 0.001.

**Figure 4:**
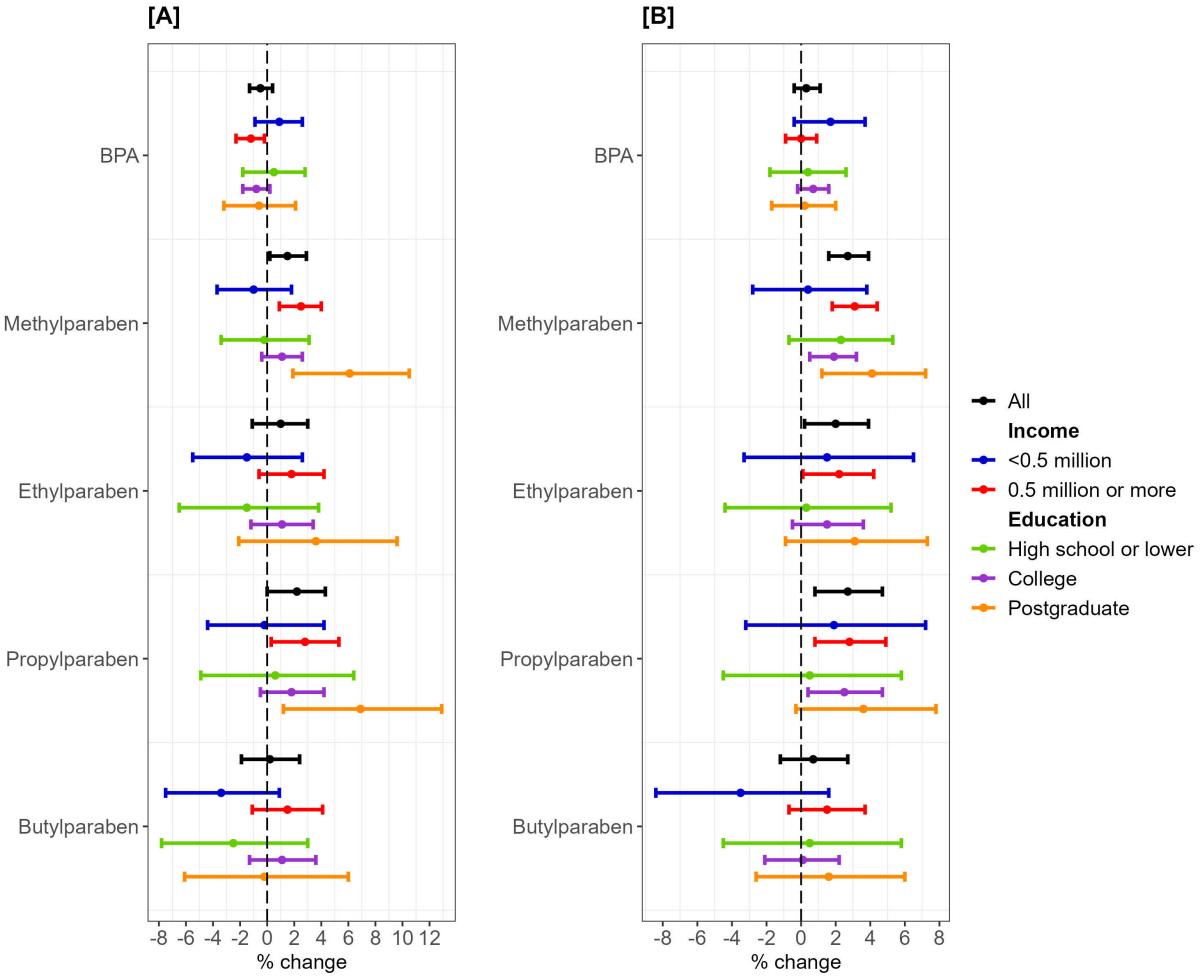
Percent change in bisphenol A/parabens urinary metabolites and use frequency of rinse-off/leave-on personal care products stratified by annual household income. One New Taiwanese Dollar (NTD) ≈ 0.031 United States Dollar. Linear regression models were adjusted for age, body mass index, working status, and geographical region. **(A)** Rinse-off products. **(B)** Leave-on products.

More frequent use of rinse-off PCPs was associated with higher concentrations of methylparaben (1.5%, 95%CI = 0.2%, 2.9%) and propylparaben (2.2%, 95%CI = 0%, 4.3%). Stratification by income maintained the direction and significance of these associations only for the above-lowest income group. More frequent use of leave-on PCPs was associated with higher concentrations of methylparaben (3.1%, 95%CI = 1.8%, 4.4%), ethylparaben (2.2%, 95%CI = 0.1%, 4.2%), and propylparaben (2.8%, 95%CI = 0.8%, 4.9%) in the above-lowest income group. Stratification by education showed the strongest associations in the postgraduate group for rinse-off PCPs with methylparaben (6.1%, 95%CI = 1.9%, 10.5%) and propylparaben (6.9%, 95%CI = 1.2%, 12.9%), and for leave-on PCPs with methylparaben (4.1%, 95%CI = 1.2%, 7.2%) (Figure 4, Supplementary Table S3).

**Figure 5:**
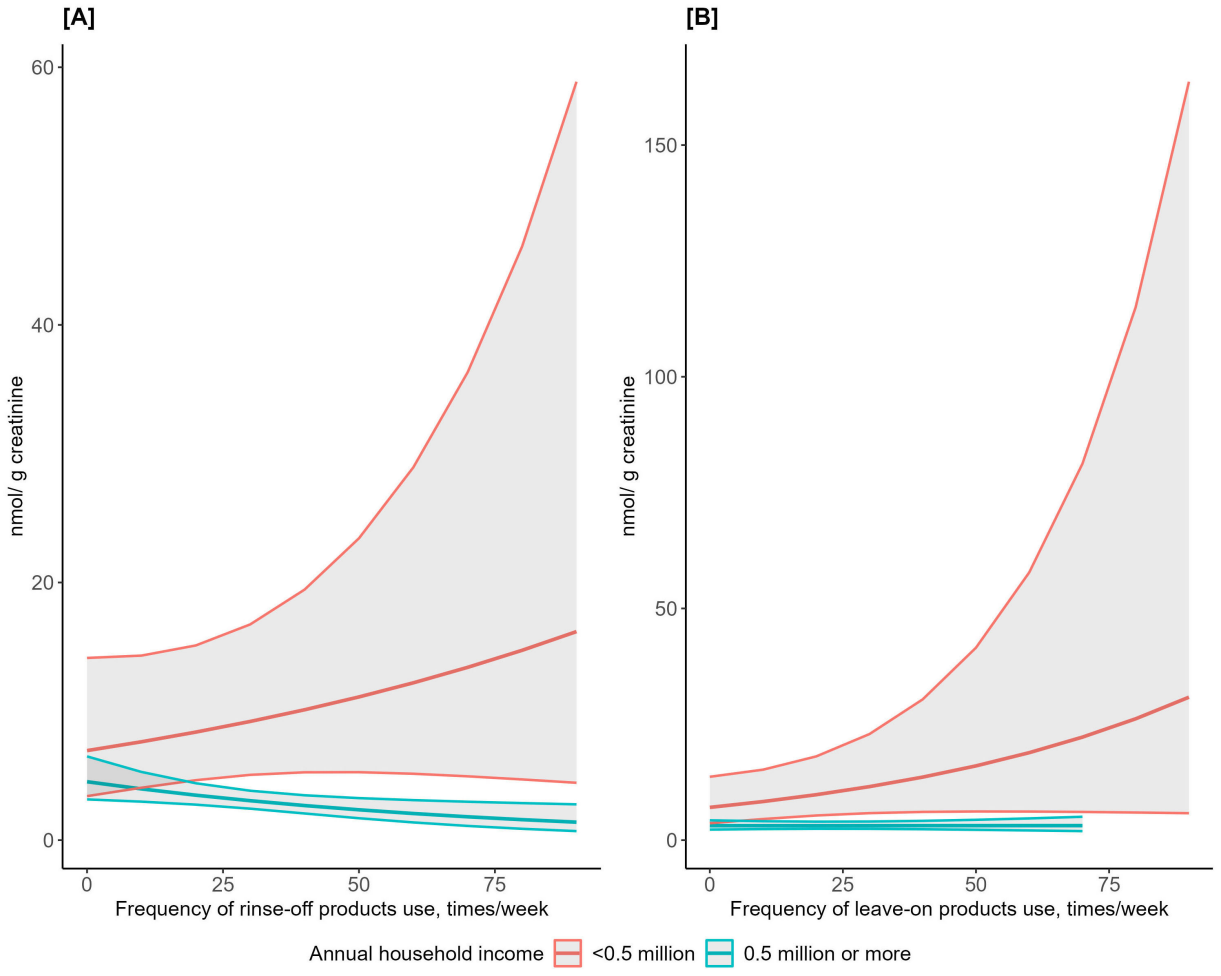
Predicted urinary concentrations of bisphenol A metabolite (nmol/g creatinine) and use frequency of rinse-off/leave-on personal care products by annual household income. One New Taiwanese Dollar (NTD) ≈ 0.031 United States Dollar. Linear regression models were adjusted for age, education, body mass index, working status, and geographical region. Plotted concentrations were exponentiated from the predicted values. Shaded areas represent 95% confidence intervals. **(A)** Rinse-off products. **(B)** Leave-on products.

Predicted BPA concentrations with 95% CI in the lowest and above-lowest income groups differed significantly for more frequent users of rinse-off and leave-on PCPs (Figure 5). Stratifications of paraben concentrations by income (Supplementary Figure S1) and of BPA/parabens by education (Supplementary Figure S2) showed overlapping 95% CI for rinse-off and leave-on PCPs.

## 4 Discussion

This study examined the role of socioeconomic status in the association between PCP use and urinary concentration of BPA/parabens in pregnant women. More frequent use of some PCPs by the study participants was associated with higher concentrations of parabens, whereas BPA concentrations were lower in the more frequent users of body wash. Paraben levels were higher in the above-lowest income group, whereas BPA levels were higher in the lowest income group, but no statistical differences were observed between the education groups. The associations between PCPs and methyl-/propylparaben were stronger in the groups with above-lowest income and postgraduate education. Significant differences in predicted BPA concentrations were observed between the two income groups for more frequent PCP use. A negative association was found between rinse-off PCP and BPA concentrations in the higher-income group, while a positive association was observed between leave-on PCP and BPA concentrations in the lowest income group.

BPA and paraben concentrations in our study were lower than those previously reported in the general population of Asian women (20, 21), which could be due to hormonal regulation, increased metabolism, and kidney function during pregnancy. When compared to pregnant women from other countries, studies with smaller sample sizes (*N* < 200) reported higher concentrations than those observed in our study (22–25). However, concentrations in our study were comparable to or higher than those reported in studies with larger sample sizes (*N* > 400) (26–29). In addition to random variation, these differences can stem from seasonal changes, climate conditions, and cultural practices. For example, exposure to benzophenone was likely to be higher during the summer months due to increased sunscreen use, while triclosan exposure could rise during peak influenza season due to more frequent handwashing (30). Cultural norms and beauty ideals, both across countries and among different ethnicities within the US, showed some differences in women's attitudes toward PCP use, their willingness to pay (31), and actual PCP use (12, 32).

Of all the analyzed PCPs, using body wash four or more times per week was associated with lower BPA levels. Aggregated evidence reported dermal contact as one of the routes for BPA exposure (4, 33). Repeated measures of BPA and different PCP use within 24 h in French women showed negative correlations with BPA for most PCPs, except for makeup remover within 6 h (34). Using body wash more frequently could reduce prolonged dermal contact with BPA and thus explain its lower levels in our study population. Positive associations with PCP frequency use were mostly found for methyl- and propylparaben, with the strongest associations for leave-on products—makeup, lotion, and toner, which was expected, since leave-on PCPs more often contain parabens in higher quantities (1). The direction of associations between methylparaben and frequency of makeup use were similar to the reported estimates from the Korean National Environmental Health Survey (35).

Frequency use of leave-on PCPs was associated with higher levels of BPA in the lowest income group with marginal statistical significance, and the predicted BPA concentrations in the lowest income group were significantly higher for leave-on/rinse-off PCPs, especially at higher frequencies of use. This finding is consistent with the previously reported higher levels of BPA in people with lower income in the analysis of National Health and Nutrition Examination Survey (NHANES) data (6). Although differences between the US and Taiwan populations exist in lifestyle, diet, study population, and culture of PCP use, pregnant women may use even more PCPs due to skin changes, self-care, and psychological or emotional reasons. Since PCPs are a less common source of BPA exposure, the difference in BPA levels between the income groups may also stem from diet or additional lifestyle factors. Higher levels of BPA found in the lowest income group of pregnant women in Taiwan warrant further monitoring of EDC exposure in the most disadvantaged populations. Interestingly, analysis of six European mother–child cohorts showed higher BPA levels in children in higher SES groups based on maternal education, employment status, and family affluence scale (50), which suggests further longitudinal investigation of our cohort.

Stratification by income showed small positive associations between leave-on/rinse-off PCPs and methyl/propylparaben in the above-lowest income group only. Higher urinary methyl- and propylparaben concentrations were also reported among high-income groups in the general American population for non-white ethnicities (36); however, a more recent analysis showed associations with ethnicity to be more consistent than those with income (8). Our findings can be explained by the less common use of PCP-containing parabens by the lowest income group, which is consistent with other studies (37).

The differences in PCP use and EDC levels between income groups in our study can be related to the observed socio-demographic characteristics. As expected, the lowest income group was younger, with lower education and employment, and predominantly resided in the East region. Notably, the distribution of BMI categories also differed by income group, with higher percentages of the underweight in the above-lowest income group and higher percentages of the overweight/obese in the lowest income group. *Post-hoc* analysis of our data revealed that underweight women in our sample were more frequent users of leave-on PCPs, including lip balm, lotion, toner, and makeup, likely reflecting aesthetic concerns (Supplementary Table S4). This finding aligns with results from the nationwide Norwegian Women and Cancer study (37). Given the high prevalence (16–18%) of underweight women entering pregnancy in Taiwan (38), further investigation into the association between weight status during pregnancy, PCP use, and EDC exposure is warranted.

Exposure to EDCs found in PCPs has been associated with multiple adverse outcomes for pregnant women and children, including high blood glucose levels, excessive weight, pubertal timing, and risk of testicular germ cell tumors (39–43). Following the precautionary principle, multiple scientific organizations advise minimizing exposure to EDCs (44). Based on the positive associations between leave-on PCPs and parabens found in our study, reducing the use of leave-on PCPs, especially makeup, during pregnancy can be recommended without adding unnecessary stress during pregnancy due to the debatable health risks of parabens (45). At the time of data collection for this study, BPA had been banned in baby bottles since 2013, and parabens have been required to be listed on cosmetic labeling since 2018 (46). Using fewer products, focusing on natural options such as coconut oil for moisturizing, and checking labels for “paraben-free” in lotions, shampoos, and cosmetics may reduce exposure to parabens and other EDCs.

Our analysis was initially guided by the previously published conceptual model that viewed race/ethnicity as a departing factor, followed by SES, which in turn influences diet, PCP use, and other behaviors resulting in EDC burden on the body (6). As Taiwan's population is more homogenous in terms of race and ethnicity compared to the US, we did not include these factors in our analysis. Mediation analysis showed mostly non-significant results, except the path from income via leave-on PCPs to methyl-/propylparaben with only a significant indirect effect (Supplementary Table S5). Previous analysis of NHANES data also showed weak or non-existent mediation between the poverty-income ratio and lifestyle or diet to EDC exposure (47), which could be due to substantial residual confounding. Although the rationale for the conceptual model is rather straightforward, adjustment for income, education, and other sociodemographic confounders (Table 3) and further stratifications suggest that income and education can also be treated as important confounders to identify more vulnerable populations. Recently published analysis of the American black women data identified groups by socioeconomic clusters jointly defined by income, education, marital status, and employment who were susceptible to mixtures of EDCs and concluded that socioeconomic status can influence exposure to EDCs (11). However, in our study population, income, education, and employment were correlated, and nearly no variability in marital status was present. Weak associations of EDC levels with income and education in this and previous analyses (13) may be due to the widespread exposure to EDCs in daily life in Taiwan and the uniform distribution of the exposure across the main island. In addition to dermal exposure through PCP use, disadvantaged populations may face greater exposure to endocrine-disrupting chemicals through ingestion and inhalation. Therefore, when investigating the environmental justice hypothesis, it is important to consider factors such as dietary patterns, food handling, indoor air pollution, and the built environment.

Although we analyzed human biomonitoring data from the nationwide birth cohort in Taiwan with a relatively large sample size, the presented results need to be interpreted within the following limitations. A potential source of bias may stem from the poor recall of the PCP use. The TMICS questionnaire generally asked about the frequency, quantity, and duration of use to capture two detailed behavior patterns; however, information on quantity and duration did not contribute to the analysis and had more missing values. More recent studies used 24- or 48-h recall to obtain information on PCP use (12, 48). Another limitation of this study is the single-spot urine measurement of BPA and parabens. In future research, repeated measurements of BPA and parabens may provide more reliable estimates of exposure. Incorporating biomarkers that reflect skin exposure, absorption, and biological impact—such as filaggrin, ceramides, cytokines, and oxidative stress markers—could enhance understanding of the effects of makeup and other leave-on PCPs on the skin. Additionally, our study population may not represent pregnant women in Taiwan, as the participants in the birth cohort were likely to be more educated and affluent; however, the comparison with national statistics did show certain similarities in age, education, and income (49). Moreover, the exclusion criteria in this cohort resulted in a relatively healthy sample. Future studies could focus on more specific populations at higher risk of exposure, such as individuals working in cosmetics sales or living near the petrochemical industries. Finally, the number of missing values on income and PCP use substantially reduced our sample size, especially compromising adequate statistical power in the subgroup analyses and resulting in a wide 95%CI, which should be interpreted with caution. Analysis of larger samples is recommended to confirm the associations in our study. The comparison between included and non-included participants showed significant differences in age and residence. Non-included participants were younger and predominantly from the South region (Supplementary Table S2). The above biases could have underestimated the real associations in our study.

This study showed that the environmental justice hypothesis may not always be supported in the context of endocrine-disrupting chemicals and income/education. While some PCPs (makeup, lotion, and toner) may be associated with parabens, which could suggest limiting their use during pregnancy, it is important not to add unnecessary stress to pregnant women during this already challenging time. Higher levels of BPA and their potential association with PCP usage frequency in the lowest income group in our study population require further investigation of sources of BPA exposure in disadvantaged populations.

## Data Availability

The datasets presented in this study can be found in online repositories. The names of the repository/repositories and accession number(s) can be found below: https://data.depositar.io/organization/about/twedcschildren.

## References

[1] Guo Y Kannan K. A survey of phthalates and parabens in personal care products from the United States and its implications for human exposure. Environ Sci Technol. (2013) 47:14442–9. 10.1021/es4042034 24261694

[2] Błedzka D Gromadzińska J Wasowicz W. Parabens. From environmental studies to human health. Environ Int. (2014) 67:27–42. 10.1016/j.envint.2014.02.007 24657492

[3] Vandenberg LN Chahoud I Heindel JJ Padmanabhan V Paumgartten FJ Schoenfelder G. Urinary, circulating, and tissue biomonitoring studies indicate widespread exposure to bisphenol A. Environ Health Perspect. (2010) 118:1055–70. 10.1289/ehp.0901716 20338858 PMC2920080

[4] von Goetz N Wormuth M Scheringer M Hungerbuhler K. Bisphenol A: how the most relevant exposure sources contribute to total consumer exposure. Risk Anal. (2010) 30:473–87. 10.1111/j.1539-6924.2009.01345.x 20136739

[5] Jala A Varghese B Dutta R Adela R Borkar RM. Levels of parabens and bisphenols in personal care products and urinary concentrations in indian young adult women: implications for human exposure and health risk assessment. Chemosphere. (2022) 297:134028. 10.1016/j.chemosphere.2022.134028 35218786

[6] Nelson JW Scammell MK Hatch EE Webster TF. Social disparities in exposures to bisphenol a and polyfluoroalkyl chemicals: a cross-sectional study within NHANES 2003-2006. Environ Health. (2012) 11:10. 10.1186/1476-069X-11-10 22394520 PMC3312862

[7] James-Todd TM Chiu YH Zota AR. Racial/ethnic disparities in environmental endocrine disrupting chemicals and women's reproductive health outcomes: epidemiological examples across the life course. Curr Epidemiol Rep. (2016) 3:161–80. 10.1007/s40471-016-0073-9 28497013 PMC5423735

[8] Nguyen VK Kahana A Heidt J Polemi K Kvasnicka J Jolliet O. A comprehensive analysis of racial disparities in chemical biomarker concentrations in United States women, 1999-2014. Environ Int. (2020) 137:105496. 10.1016/j.envint.2020.105496 32113086 PMC7137529

[9] Raley E Quirós-Alcalá L Matsui EC. Chemical exposures via personal care products and the disproportionate asthma burden among the U.S. Black Population. J Allergy Clin Immunol Pract. (2021) 9:3290–2. 10.1016/j.jaip.2021.04.063 33975033 PMC8434946

[10] Bloom MS Wenzel AG Brock JW Kucklick JR Wineland RJ Cruze L. Racial disparity in maternal phthalates exposure; association with racial disparity in fetal growth and birth outcomes. Environ Int. (2019) 127:473–86. 10.1016/j.envint.2019.04.005 30981018

[11] Schildroth S Bethea TN Wesselink AK Friedman A Fruh V Calafat AM. Personal care products, socioeconomic status, and endocrine-disrupting chemical mixtures in Black Women. Environ Sci Technol. (2024) 58:3641–53. 10.1021/acs.est.3c06440 38347750

[12] Preston EV Chan M Nozhenko K Bellavia A Grenon MC Cantonwine DE. Socioeconomic and racial/ethnic differences in use of endocrine-disrupting chemical-associated personal care product categories among pregnant women. Environ Res. (2021) 198:111212. 10.1016/j.envres.2021.111212 33957140 PMC8886956

[13] Waits A Chang C-H Huang Y-F Tsai M-S Hou J-W Wang P-W. Income inequalities in exposure to endocrine disrupting chemicals among pregnant women in Taiwan. Environmental Advances. (2024) 15. 10.1016/j.envadv.2023.100470

[14] Wu CF Chen HM Sun CW Chen ML Hsieh CJ Wang SL. Cohort profile: the taiwan maternal and infant cohort study (TMICS) of phthalate exposure and health risk assessment. Int J Epidemiol. (2018) 47:1047–1047j. 10.1093/ije/dyy067 29718277

[15] Tan K. Appropriate body-mass index for Asian populations and its implications for policy and intervention strategies. Lancet. (2004) 63:157–63. 10.1016/S0140-6736(03)15268-3 14726171

[16] Taiwan Health Promotion Administration (2018). BMI Classification (in Chinese). http://health99.hpa.gov.tw/OnlinkHealth/Onlink_BMI.aspx (accessed September 20, 2024).

[17] Chang CH Huang YF Wang PW Lai CH Huang LW Chen HC. Associations between prenatal exposure to bisphenol a and neonatal outcomes in a Taiwanese cohort study: mediated through oxidative stress? Chemosphere. (2019) 226:290–7. 10.1016/j.chemosphere.2019.03.093 30933738

[18] Chang CH Wang PW Liang HW Huang YF Huang LW Chen HC. The sex-specific association between maternal paraben exposure and size at birth. Int J Hyg Environ Health. (2019) 222:955–64. 10.1016/j.ijheh.2019.06.004 31248753

[19] Hornung RW Reed LD. Estimation of average concentration in the presence of nondetectable values. Appl Occup Environ Hyg. (1990) 5:46–51. 10.1080/1047322X.1990.10389587

[20] Cui FP Yang P Liu C Chen PP Deng YL Miao Y. Urinary bisphenol A and its alternatives among pregnant women: predictors and risk assessment. Sci Total Environ. (2021) 784:147184. 10.1016/j.scitotenv.2021.147184 33901963

[21] Huang PC Chen HC Chou WC Lin HW Chang WT Chang JW. Cumulative risk assessment and exposure characteristics of parabens in the general Taiwanese using multiple hazard indices approaches. Sci Total Environ. (2022) 843:156821. 10.1016/j.scitotenv.2022.156821 35738379

[22] Kang S., Kim, S., Park, J., Kim, H. J., Lee, J., Choi, G., et al. (2013). Urinary paraben concentrations among pregnant women and their matching newborn infants of korea, and the association with oxidative stress biomarkers. Sci Total Environ 461-462: 214–221. 10.1016/j.scitotenv.2013.04.097 23727995

[23] Casas M Forns J Martínez D Avella-García C Valvi D Ballesteros-Gómez A. Exposure to bisphenol A during pregnancy and child neuropsychological development in the INMA-Sabadell cohort. Environ Res. (2015) 142:671–9. 10.1016/j.envres.2015.07.024 26343751

[24] Moos RK Apel P Schroter-Kermani C Kolossa-Gehring M Bruning T Koch HM. Daily intake and hazard index of parabens based upon 24 h urine samples of the German environmental specimen bank from 1995 to 2012. J Expo Sci Environ Epidemiol. (2017) 27:591–600. 10.1038/jes.2016.65 27901017

[25] Hajizadeh Y Kiani Feizabadi G Ebrahimpour K Shoshtari-Yeganeh B Fadaei S Darvishmotevalli M. Urinary Paraben Concentrations and Their Implications for Human exposure in iranian pregnant women. Environ Sci Pollut Res Int. (2020) 27:14723–34. 10.1007/s11356-020-07991-2 32052325

[26] Wu C Huo W Li Y Zhang B Wan Y Zheng T. Maternal urinary paraben levels and offspring size at birth from a Chinese birth cohort. Chemosphere. (2017) 172:29–36. 10.1016/j.chemosphere.2016.12.131 28061343

[27] Aker AM Johns L McElrath TF Cantonwine DE Mukherjee B Meeker JD. Associations between maternal phenol and paraben urinary biomarkers and maternal hormones during pregnancy: a repeated measures study. Environ Int. (2018) 113:341–9. 10.1016/j.envint.2018.01.006 29366524 PMC5866216

[28] Wen Q Zhou Y Wang Y Li J Zhao H Liao J. Association between urinary paraben concentrations and gestational weight gain during pregnancy. J Expo Sci Environ Epidemiol. (2020) 30:845–55. 10.1038/s41370-020-0205-7 32042059

[29] Lan L Wan Y Qian X Wang A Mahai G He Z. Urinary paraben derivatives in pregnant women at three trimesters: variability, predictors, and association with oxidative stress biomarkers. Environ Int. (2022) 165:107300. 10.1016/j.envint.2022.107300 35635959

[30] Romano ME Kalloo G Etzel T Braun JM. Seasonal variation in exposure to endocrine-disrupting chemicals. Epidemiology. (2017) 28:e42–3. 10.1097/EDE.0000000000000696 28570386 PMC5539945

[31] Madan S Basu S Ng S Ching Lim EA. Impact of culture on the pursuit of beauty: evidence from five countries. J Int Market. (2018) 26:54–68. 10.1177/1069031X18805493

[32] Wang VA Chu MT Chie L Gaston SA Jackson CL Newendorp N. Acculturation and endocrine disrupting chemical-associated personal care product use among us-based foreign-born chinese women of reproductive age. J Expo Sci Environ Epidemiol. (2021) 31:224–32. 10.1038/s41370-020-00279-0 33235331 PMC7954893

[33] Lu S Yu Y Ren L Zhang X Liu G Yu Y. Estimation of intake and uptake of bisphenols and triclosan from personal care products by dermal contact. Sci Total Environ. (2018) 621:1389–96. 10.1016/j.scitotenv.2017.10.088 29054660

[34] Nakiwala D Vernet C Lyon-Caen S Lavorel A Rolland M Cracowski C. Use of personal care products during pregnancy in relation to urinary concentrations of select phenols: a longitudinal analysis from the sepages feasibility study. Int J Hyg Environ Health. (2020) 227:113518. 10.1016/j.ijheh.2020.113518 32279061 PMC8449543

[35] Lim S. The associations between personal care products use and urinary concentrations of phthalates, parabens, and triclosan in various age groups: the Korean national environmental health survey cycle 3 2015-2017. Sci Total Environ. (2020) 742:140640. 10.1016/j.scitotenv.2020.140640 32721747

[36] Belova A Greco SL Riederer AM Olsho LE Corrales MA. A method to screen us environmental biomonitoring data for race/ethnicity and income-related disparity. Environ Health. (2013) 12:114. 10.1186/1476-069X-12-114 24354733 PMC3893603

[37] Aniansson B Veierød MB Rylander C Lund E Sandanger TM. Characterization of heavy users of skin care products among Norwegian women from 2003 to 2011. Arch Public Health. (2016) 74:53. 10.1186/s13690-016-0165-5 28018591 PMC5165705

[38] Waits A Guo C-Y Chien L-Y. Inadequate gestational weight gain contributes to increasing rates of low birth weight in Taiwan: 2011–2016 Nationwide Surveys. Taiwan J Obstet Gynecol. (2021) 60:857–62. 10.1016/j.tjog.2021.07.013 34507661

[39] Ghazarian AA Trabert B Robien K Graubard BI McGlynn KA. Maternal use of personal care products during pregnancy and risk of testicular germ cell tumors in sons. Environ Res. (2018) 164:109–13. 10.1016/j.envres.2018.02.017 29482183 PMC5911208

[40] Bellavia A Minguez-Alarcon L Ford JB Keller M Petrozza J Williams PL. Association of self-reported personal care product use with blood glucose levels measured during pregnancy among women from a fertility clinic. Sci Total Environ. (2019) 695:133855. 10.1016/j.scitotenv.2019.133855 31421341 PMC6868339

[41] Harley KG Berger KP Kogut K Parra K Lustig RH Greenspan LC. Association of phthalates, parabens and phenols found in personal care products with pubertal timing in girls and boys. Hum Reprod. (2019) 34:109–17. 10.1093/humrep/dey337 30517665 PMC6295961

[42] Leppert B Strunz S Seiwert B Schlittenbauer L Schlichting R Pfeiffer C. Maternal paraben exposure triggers childhood overweight development. Nat Commun. (2020) 11:561. 10.1038/s41467-019-14202-1 32047148 PMC7012887

[43] Højsager FD Kyhl HB Frederiksen H Juul A Andersson AM Andersen MS. Prenatal exposure to butyl paraben is associated with fat percentage in 7-year-old boys. J Clin Endocrinol Metab. (2021) 106:e2633–8. 10.1210/clinem/dgab167 33720358

[44] Wong KH Durrani TS. Exposures to endocrine disrupting chemicals in consumer products-a guide for pediatricians. Curr Probl Pediatr Adolesc Health Care. (2017) 47:107–18. 10.1016/j.cppeds.2017.04.002 28526231

[45] Fransway AF Fransway PJ Belsito DV Yiannias JA. Paraben toxicology. Dermatitis. (2019) 30:32–45. 10.1097/DER.0000000000000428 30570577

[46] Ministry of Health and Welfare (2018). Cosmetic Hygiene and Safety Act. Available online at: https://law.moj.gov.tw/ENG/LawClass/LawAll.aspx?pcode=L0030013 (accessed September 20, 2024).

[47] Tyrrell J Melzer D Henley W Galloway TS Osborne NJ. Associations between socioeconomic status and environmental toxicant concentrations in adults in the USA: NHANES 2001-2010. Environ Int. (2013) 59:328–35. 10.1016/j.envint.2013.06.017 23892225

[48] González-Alzaga B Hernández AF Kim Pack L Iavicoli I Tolonen H Santonen T. The questionnaire design process in the european human biomonitoring initiative (hbm4eu). Environ Int. (2022) 160:107071. 10.1016/j.envint.2021.107071 34979351

[49] Taiwan Statistical Bureau (2013). Social Indicators, https://eng.stat.gov.tw/News_Content.aspx?n=2386&s=214596 (accessed September 20, 2024).

[50] Montazeri P Thomsen C Casas M de Bont J Haug LS Maitre L. Socioeconomic position and exposure to multiple environmental chemical contaminants in six European mother-child cohorts. Int J Hyg Environ Health. (2019) 222:864–72. 10.1016/j.ijheh.2019.04.002 31010791 PMC8713641

