# Supplementary material for: Socioeconomic status in the association between use of personal care products and exposure to endocrine-disrupting chemicals in pregnant Taiwanese women
Source: Front Public Health. 2025 Apr 10;13:1537669. doi: 10.3389/fpubh.2025.1537669 (PMC12019293; doi:10.3389/fpubh.2025.1537669)

**Socioeconomic status in the associations between use of personal care products and exposure to endocrine-disrupting chemicals in pregnant Taiwanese women**

Supplementary materials

Table S1. Standardization of the questionnaire response on frequency use of personal care products.

| How often do you use [*product type*]? | Original data set values | Standardized values, times per week |
| --- | --- | --- |
| Never | 0 | 0 |
| 1-3 times per month | 1 | 0.5 |
| 1-3 times per week | 2 | 2 |
| 4-6 times per week | 3 | 5 |
| Once per day | 4 | 7 |
| Twice per day | 5 | 14 |
| Trice or more per day | 6 | 21 |

Table S2. Missingness analysis of annual household income/personal care products (PCP) use by age and region.

|  | **No missing income/PCP** | **Missing income/PCP** | **P-value** |
| --- | --- | --- | --- |
|  | **(n=677)** | **(n=506)** |  |
| **Age, years** | Mean (SD) | Mean (SD) |  |
|  | 32.2 (4.3) | 30.0 (4.9) | <0.001 |
| **Region** | n (%) | n (%) |  |
| Central | 317 (34.3%) | 57 (22.1%) | <0.001 |
| East | 259 (28.1%) | 23 (9.4%) |  |
| North | 189 (20.5%) | 36 (15.2%) |  |
| South | 157 (17.0%) | 137 (53.3%) |  |

Table S3. Associations between rinse-off/leave-on personal care products and endocrine-disrupting chemicals stratified by annual household income.

|  |  |  | Parabens | | | |
| --- | --- | --- | --- | --- | --- | --- |
|  |  | **Bisphenol A** | **Methylparaben** | **Ethylparaben** | **Propylparaben** | **Butylparaben** |
|  |  | % change (95% CI) | % change (95% CI) | % change (95% CI) | % change (95% CI) | % change (95% CI) |
| Rinse-off products | |  |  |  |  |  |
| Frequency of use, times/week | All | -0.5 (-1.3, 0.4) | **1.5 (0.2, 2.9) ^*^** | 1.0 (-1.1, 3) | **2.2 (0, 4.3) ^*^** | 0.2 (-1.9, 2.4) |
| Annual household income, NTD^1^ | <0.5 million | 0.9 (-0.9, 2.6) | -1.0 (-3.7, 1.8) | -1.5 (-5.5, 2.6) | -0.2 (-4.4, 4.2) | -3.4 (-7.5, 0.9) |
|  | ≥0.5 million | **-1.2 (-2.3, -0.2) ^*^** | **2.5 (0.9, 4.0) ^**^** | 1.8 (-0.6, 4.2) | **2.8 (0.3, 5.3) ^*^** | 1.5 (-1.1, 4.1) |
| Education | High school or lower | 0.5 (-1.8, 2.8) | -0.2 (-3.4, 3.1) | -1.5 (-6.5, 3.8) | 0.6 (-4.9, 6.4) | -2.5 (-7.8, 3) |
|  | College | -0.8 (-1.8, 0.2) | 1.1 (-0.4, 2.6) | 1.1 (-1.2, 3.4) | 1.8 (-0.5, 4.2) | 1.1 (-1.3, 3.6) |
|  | Postgraduate | -0.6 (-3.2, 2.1) | **6.1 (1.9, 10.5) ^**^** | 3.6 (-2.1, 9.6) | **6.9 (1.2, 12.9) ^*^** | -0.2 (-6.1, 6) |
| Leave-on products | |  |  |  |  |  |
| Frequency of use, times/week | All | 0.3 (-0.4, 1.1) | **2.7 (1.6, 3.9) ^***^** | 2.0 (0.2, 3.9) | **2.7 (0.8, 4.7) ^**^** | 0.7 (-1.2, 2.7) |
| Annual household income, NTD^1^ | <0.5 million | *1.7 (-0.4, 3.7)* | 0.4 (-2.8, 3.8) | 1.5 (-3.3, 6.5) | 1.9 (-3.2, 7.2) | -3.5 (-8.4, 1.6) |
|  | ≥0.5 million | 0 (-0.9, 0.9) | **3.1 (1.8, 4.4) ^***^** | **2.2 (0.1, 4.2) ^*^** | **2.8 (0.8, 4.9) ^**^** | 1.5 (-0.7, 3.7) |
| Education | High school or lower | 0.4 (-1.8, 2.6) | 2.3 (-0.7, 5.3) | 0.3 (-4.4, 5.2) | 0.5 (-4.5, 5.8) | 0.5 (-4.5, 5.8) |
|  | College | 0.7 (-0.2, 1.6) | **1.9 (0.5, 3.2) ^**^** | 1.5 (-0.5, 3.6) | **2.5 (0.4, 4.7) ^*^** | 0.1 (-2.1, 2.2) |
|  | Postgraduate | 0.2 (-1.7, 2) | **4.1 (1.2, 7.2) ^**^** | 3.1 (-0.9, 7.3) | 3.6 (-0.3, 7.8) | 1.6 (-2.6, 6) |

^1^ One New Taiwanese Dollar (NTD) ≈ 0.031 United States Dollar

Linear regression models were adjusted for age, education, body-mass index, working status and geographical region.

^*^ p<0.05, ^**^ p<0.01, ^***^ p<0.001

Table S4. Distribution of personal care products use across body-mass index categories.

|  | Underweight | Normal | Overweight | Obese | P-value |
| --- | --- | --- | --- | --- | --- |
|  | **(N=90)** | **(N=432)** | **(N=79)** | **(N=57)** |  |
| Use of personal care products use, times/week, mean (SD) | | | |  |  |
| Total, range 0-66 | 55.2 (26.1) | 45.7 (23.3) | 47.4 (21.6) | 40.5 (28.2) | **0.006** |
| Rinse-off products, range 0-24 | 28.3 (12.5) | 26.0 (13.1) | 29.8 (15.4) | 27.0 (17.4) | 0.115 |
| Leave-on products, range 0-42 | 26.9 (17.9) | 19.7 (15.2) | 17.5 (14.0) | 13.5 (16.9) | **<0.001** |
| Categorized use of personal care products, n (%) | | |  |  |  |
| Body wash |  |  |  |  |  |
| < 4 times/week | 16 (17.8%) | 94 (21.8%) | 14 (17.7%) | 11 (19.3%) | 0.740 |
| > 3 times/week | 74 (82.2%) | 338 (78.2%) | 65 (82.3%) | 46 (80.7%) |  |
| Shampoo |  |  |  |  |  |
| < 4 times/week | 25 (27.8%) | 112 (25.9%) | 11 (13.9%) | 12 (21.1%) | 0.104 |
| > 3 times/week | 65 (72.2%) | 320 (74.1%) | 68 (86.1%) | 45 (78.9%) |  |
| Facial cleanser |  |  |  |  |  |
| < 4 times/week | 5 (5.6%) | 57 (13.2%) | 11 (13.9%) | 12 (21.1%) | 0.051 |
| > 3 times/week | 85 (94.4%) | 375 (86.8%) | 68 (86.1%) | 45 (78.9%) |  |
| Hand soap |  |  |  |  |  |
| < 4 times/week | 42 (46.7%) | 226 (52.3%) | 30 (38.0%) | 28 (49.1%) | 0.119 |
| > 3 times/week | 48 (53.3%) | 206 (47.7%) | 49 (62.0%) | 29 (50.9%) |  |
| Lotion |  |  |  |  |  |
| < 4 times/week | 19 (21.1%) | 141 (32.6%) | 25 (31.6%) | 32 (56.1%) | **<0.001** |
| > 3 times/week | 71 (78.9%) | 291 (67.4%) | 54 (68.4%) | 25 (43.9%) |  |
| Toner |  |  |  |  |  |
| < 4 times/week | 27 (30.0%) | 158 (36.6%) | 32 (40.5%) | 32 (56.1%) | **0.012** |
| > 3 times/week | 63 (70.0%) | 274 (63.4%) | 47 (59.5%) | 25 (43.9%) |  |
| Lip balm |  |  |  |  |  |
| < 4 times/week | 48 (53.3%) | 299 (69.2%) | 56 (70.9%) | 46 (80.7%) | **0.003** |
| > 3 times/week | 42 (46.7%) | 133 (30.8%) | 23 (29.1%) | 11 (19.3%) |  |
| Makeup |  |  |  |  |  |
| < 4 times/week | 48 (53.3%) | 283 (65.5%) | 63 (79.7%) | 47 (82.5%) | **<0.001** |
| > 3 times/week | 42 (46.7%) | 149 (34.5%) | 16 (20.3%) | 10 (17.5%) |  |
| Essential oil |  |  |  |  |  |
| never | 78 (86.7%) | 379 (87.7%) | 67 (84.8%) | 52 (91.2%) | 0.723 |
| ever | 12 (13.3%) | 53 (12.3%) | 12 (15.2%) | 5 (8.8%) |  |
| Perfume |  |  |  |  |  |
| never | 70 (77.8%) | 358 (82.9%) | 68 (86.1%) | 40 (70.2%) | 0.063 |
| ever | 20 (22.2%) | 74 (17.1%) | 11 (13.9%) | 17 (29.8%) |  |
| Hair spray |  |  |  |  |  |
| never | 84 (93.3%) | 401 (92.8%) | 71 (89.9%) | 52 (91.2%) | 0.789 |
| ever | 6 (6.7%) | 31 (7.2%) | 8 (10.1%) | 5 (8.8%) |  |

Table S5. Mediation analysis of use frequency of personal care products in the association between income/education and bisphenol A/parabens urinary metabolites.

| Exposure | Mediator | Outcome | Direct effect | Indirect effect | % mediated |
| --- | --- | --- | --- | --- | --- |
| Socioeconomic factor | **Personal care products** | **Metabolite** |  |  |  |
| Income | **Rinse-off** | Bisphenol A | 0.0 (-0.328, 0.334) | -0.012 (-0.051, 0.012) | 0.5 |
|  |  | Methylparaben | 0.276 (-0.227, 0.791) | 0.030 (-0.019, 0.104) | 6.4 |
|  |  | Ethylparaben | 0.248 (-0.500, 1.013) | 0.016 (-0.033, 0.091) | 1.3 |
|  |  | Propylparaben | 0.224 (-0.534, 0.999) | 0.038 (-0.025, 0.139) | 4.7 |
|  |  | Butylparaben | 0.536 (-0.253, 1.343) | 0.003 (-0.056, 0.068) | 0.2 |
|  | **Leave-on** | Bisphenol A | -0.036 (-0.364, 0.301) | 0.023 (-0.014, 0.074) | 1.3 |
|  |  | Methylparaben | 0.204 (-0.297, 0.718) | 0.102 (0.016, 0.220) ^*^ | 25.7 |
|  |  | Ethylparaben | 0.194 (-0.555, 0.962) | 0.070 (-0.011, 0.196) | 10.8 |
|  |  | Propylparaben | 0.153 (-0.605, 0.930) | 0.108 (0.009, 0.258) **^*^** | 18.2 |
|  |  | Butylparaben | 0.520 (-0.272, 1.331) | 0.019 (-0.077, 0.126) | 2.1 |
| Education | **Rinse-off** | Bisphenol A | -0.001 (-0.035, 0.031) | -0.2 (-0.586, 0.195) | 0.3 |
|  |  | Methylparaben | 0.003 (-0.067, 0.077) | 0.098 (-0.497, 0.707) | 0.9 |
|  |  | Ethylparaben | 0.002 (-0.054, 0.063) | 0.692 (-0.190, 1.593) | 0.1 |
|  |  | Propylparaben | 0.004 (-0.087, 0.100) | 0.266 (-0.630, 1.181) | 0.5 |
|  |  | Butylparaben | 0.001 (-0.047, 0.051) | 0.856 (-0.074, 1.807) | 0 |
|  | **Leave-on** | Bisphenol A | 0.022 (-0.012, 0.076) | -0.223 (-0.611, 0.173) | 5.6 |
|  |  | Methylparaben | 0.095 (-0.004, 0.228) | 0.006 (-0.587, 0.611) | 17.4 |
|  |  | Ethylparaben | 0.061 (-0.018, 0.193) | 0.632 (-0.253, 1.534) | 6.9 |
|  |  | Propylparaben | 0.099 (-0.007, 0.262) | 0.171 (-0.727, 1.086) | 12.8 |
|  |  | Butylparaben | 0.017 (-0.074, 0.125) | 0.839 (-0.096, 1.793) | 1.3 |

Linear regression models were adjusted for age, education, body-mass index, working status, and geographical region.

^*^ p<0.05

Figure S1. Associations between parabens urinary concentrations (nmol/g creatinine) and frequency score of personal care products use by annual household income. One New Taiwanese Dollar (NTD) ≈ 0.031 United States Dollar. Linear regression models were adjusted for age, education, body-mass index, working status and geographical region. Plotted concentrations were exponentiated from the predicted values. Shaded areas represent 95% confidence intervals.


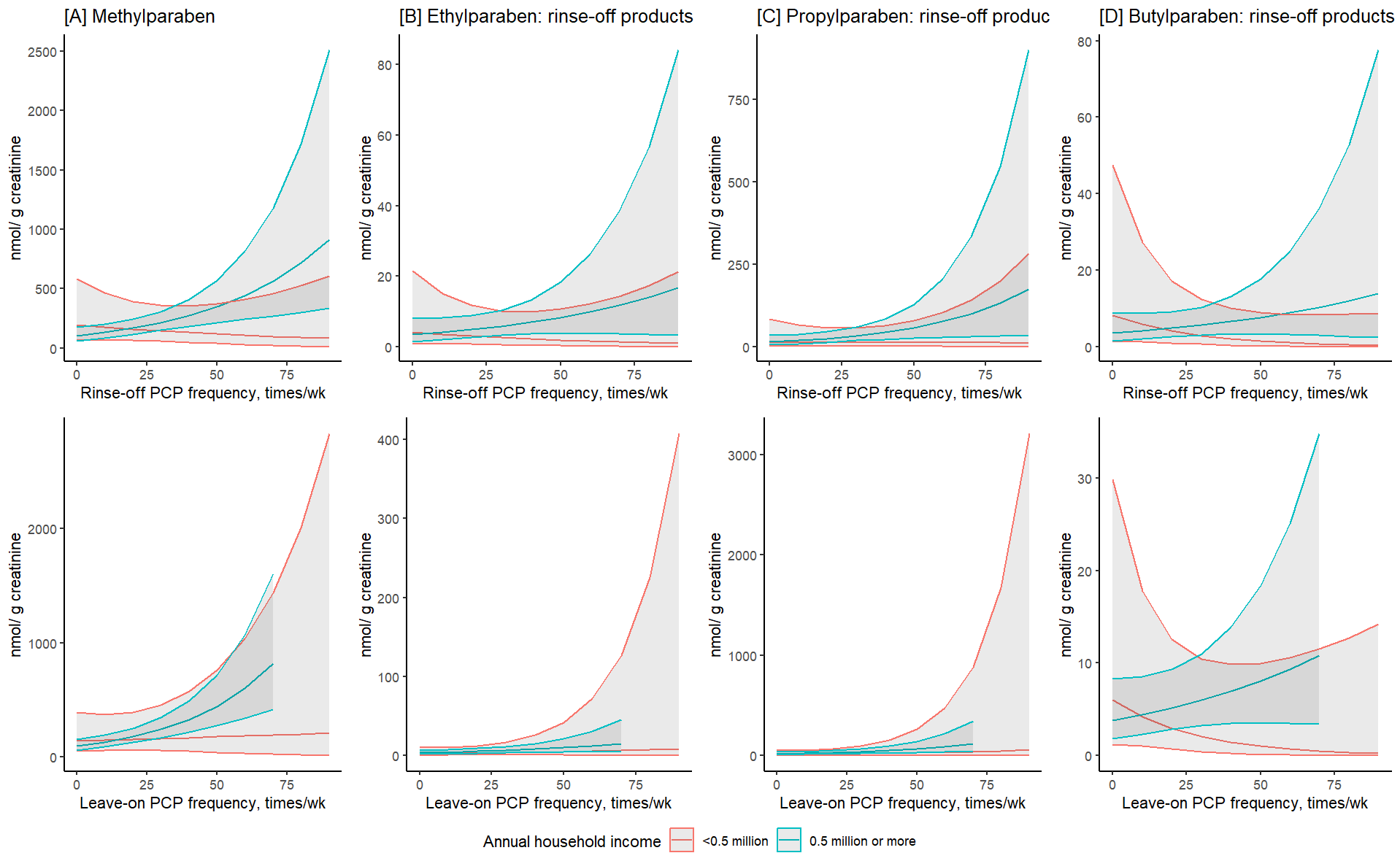


Figure S2. Associations between parabens urinary concentrations (nmol/g creatinine) and frequency score of personal care products use by education level. Linear regression models were adjusted for age, income, body-mass index, working status and geographical region. Plotted concentrations were exponentiated from the predicted values. Shaded areas represent 95% confidence intervals.


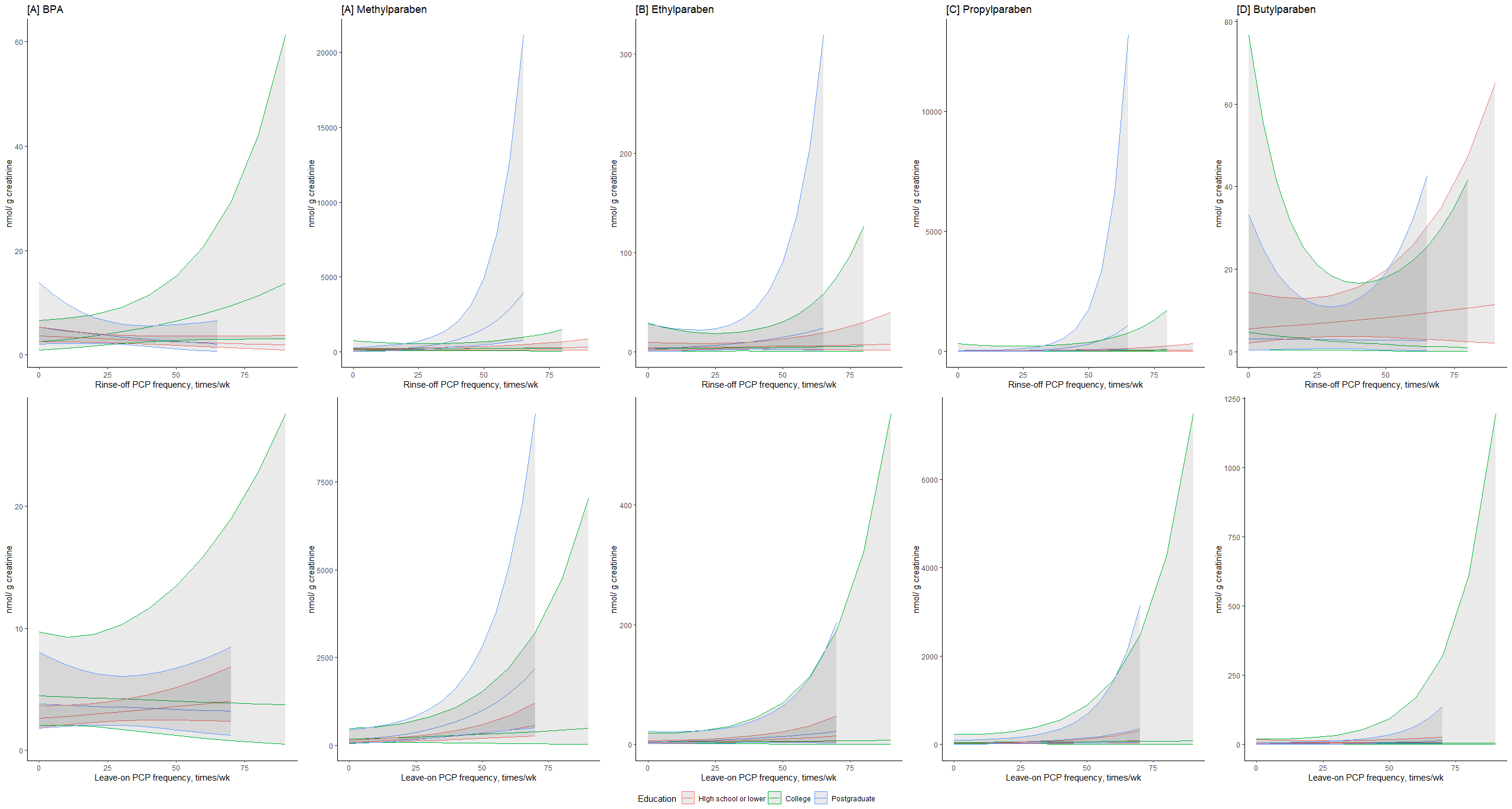

Supplement: Supplementary file 1 [file Data_Sheet_1.docx]
